# Removing Organic Matter and Nutrients from Pig Farm Wastewater with a Constructed Wetland System

**DOI:** 10.3390/ijerph15051031

**Published:** 2018-05-21

**Authors:** Celia De La Mora-Orozco, Irma Julieta González-Acuña, Ruben Alfonso Saucedo-Terán, Hugo Ernesto Flores-López, Hector Osbaldo Rubio-Arias, Jesús Manuel Ochoa-Rivero

**Affiliations:** 1 National Research Institute for Forestry, Agriculture and Animal Production, Av. Biodiversidad 2470, Tepatitlán de Morelos 47600, Jalisco, Mexico; flores.hugo@inifap.gob.mx; 2 National Research Institute for Forestry, Agriculture and Animal Production, Km 6 Entronque Carretera Internacional Mexico-Nogales, Santiago Ixcuintla 63300, Nayarit, Mexico; gonzalez.irmajulieta@inifap.gob.mx; 3 Retired, Former Researcher from the National Research Institute for Forestry, Agriculture and Animal Production, Aldama Chihuahua 27440, Mexico; rasteran@yahoo.com.mx; 4 Department of Natural Resources, Autonomous University of Chihuahua, Mexico. Periférico Francisco R. Almada, Km. 1. Facultad de Zootecnia, Chihuahua 33820, Mexico; rubio05@hotmail.com; 5 National Research Institute for Forestry, Agriculture and Animal Production, Km. 33.3 Carr. Chihuahua-Ojinaga, Cd. Aldama, Chihuahua 27440, Mexico; ochoa.jesus@inifap.gob.mx

**Keywords:** pig farm wastewater, organic matter, nutrient removal, surface and subsurface wetland

## Abstract

Pollutants from pig farms in Mexico have caused problems in many surface water reservoirs. Growing concern has driven the search for low-cost wastewater treatment solutions. The objective of this research was to evaluate the potential of an in-series constructed wetland to remove nutrients from wastewater from a pig farm. The wetland system had a horizontal flow that consisted of three cells, the first a surface water wetland, the second a sedimentation cell, and the third a subsurface flow wetland. The vegetation used was *Thypa* sp. and *Scirpus* sp. A mix of soil with red volcanic rock (10–30 mm diameter) and yellow sand (2–8 mm diameter) was used as a substrate for the vegetation. The experiments were carried out in duplicate. Water samples were collected at the inflow and outflow of the cells. Two hydraulic retention times (HRT) (5 and 10 days) and three treatments were evaluated: 400, 800, and 1200 mg·L^−1^ of chemical oxygen demand (COD) concentration. Data was collected in situ for temperature, pH, dissolved oxygen (DO), electrical conductivity (EC), and total dissolved solids (TDS). COD, total Kjeldahl nitrogen (TKN), ammonia nitrogen (NH_3_–N), and total phosphorous (TP) were analyzed in the laboratory. The results showed that the in-series constructed wetland is a feasible system for nutrient pollutant removal, with COD removal efficiency of 76% and 80% mg·L^−1^ for a 5- and 10-day HRT, respectively. The removal efficiency for TKN, NH_3_–N, and TP reached about 70% with a 5-day HRT, while a removal of 85% was obtained with a 10-day HRT. The wetland reached the maximum removal efficiency with a 10-day HRT and an inflow load of 400 mg·L^−1^ of organic matter. The results indicate that HRT positively affects removal efficiency of COD and TDS. On the other hand, the HRT was not the determining factor for TP removal. Treatment one, with an initial COD concentration of 400 mg·L^−1^, had the highest removal of the assessed pollutants, allowing for the use of water for irrigation according to Mexican regulatory standards (NOM-001). The water quality resulting from treatments two and three (T2 = 800 mg·L^−1^ of COD and T3 = 1200 mg·L^−1^ of COD) did not comply with minimal requirements for irrigation water.

## 1. Introduction

According to the Mexican agriculture, livestock and forestry census [1], pig farming is the third most important livestock activity in Mexico, with a population of 13.020 million animals. Although pig farming is conducted throughout the country, it is mainly concentrated in the central and southeastern regions [2]. Waste from pig farming, which includes water used to clean sties, feces and urine, and food residue, has had a negative impact on the environment. Water quality degradation poses risks to the environment and public health, because of which significant attention is being directed to developing cost-effective technologies to remove organic matter and nutrients, especially nitrogen and phosphorous, from wastewater [3,4,5]. However, many of these technologies involve high financial and energy costs. Different methodologies have been experimented to remove contaminants from wastewater, such as using metakaolin geopolymer (ion-exchange) which was obtained by geopolymerization/granulation [6]. This material has been used to remove NH_4_ from synthetic and domestic wastewater, resulting in maximum exchange capacities of 31.79, 28.77, and 17.75 mg·g^−1^ in synthetic, screened, and pre-sedimented municipal wastewater, respectively. Different anaerobic systems have also been investigated, involving polypropylene beads, sponge cubes, and coconut fibers. A chemical oxygen demand (COD) removal efficiency of 80% was obtained from pig farm wastewater [7]. An experiment in North Carolina, USA investigated the use of organic polymers (polyacrylamide) to separate solids by flocculation and reduce carbon compounds in pig farm wastewater. The results indicated a maximum 20% of solids can be removed just by filtering. However, when using polyacrylamide before filtering, 95% efficiency was obtained for total suspended solids (TSS), 69% for suspended volatile solids (SVS), 69% for COD, and 59% for five-day biochemical oxygen demand (BOD_5_). Polyacrylamide also improves the reduction of organic P and N [8]. Other systems have also been experimented with, such as vermifiltration, with reported COD and total Kjeldahl nitrogen (TKN) removal efficiency of 70% [9,10]. Vermifiltration uses earthworms as the biological medium, which feed on contaminants in wastewater [9,11].

Some researchers have proposed constructed wetlands as a feasible alternative for reducing nutrient concentrations in wastewater through removal pathways like microbial biomass growth and direct vegetation uptake [12,13]. Some water-borne pollutants can be transformed into less harmful substances by wetland biota that use pollutants as nutrients [14]. Another wetland mitigation mechanism is sedimentation, through which pollutants are eliminated from water by their adherence to particulate matter [15,16]. According to other researchers [17], natural wetlands reach nitrogen removal efficiency as high as 77%, while constructed wetlands attain removal efficiencies of up to 44%. Other authors [18] reported removal efficiency in constructed wetlands of 18–27% for nitrogen and 40–81% for phosphorous. Similarly, other authors [19] observed nitrates (NO_3_–N) removal efficiency at about 31–42% in constructed wetlands and noticed that sediment absorption and vegetation uptake were the principal factors responsible for decreased contamination [19]. On the other hand, hydraulic retention time is a physical factor strongly associated with the effectiveness of nutrient removal, which can be characterized by water retention time experiments. According to other researchers [20], the removal capacity of this type of purification system is due to the combined effects of hydraulic retention time, the filtering action of the substrate, and biological activity. There are reports of constructed wetlands used as tertiary treatment (using *Phragmites australis*) to remove N, P, and COD from wastewater from pig farms in Belgium. A subsurface flow wetland was set up and different substrates were assessed, including sand, loam, clayey sand, and expanded clay (argex). Removal efficiency varied according to the substrate, ranging from 64% to 75% for COD, 73% to 83% for N, and 71% to 92% for P. Nevertheless, the levels in the effluent were significantly above maximum acceptable levels according to Belgian standards of 2 mg·L^−1^, 15 mg·L^−1^, and 125 mg·L^−1^ for P, N, and COD, respectively [21]. Years later, the same authors reported that in the first stage of the experiment, they did not reach the official Belgian standards. However, after optimizing the system, they were able to reach levels that complied with Belgian standards for effluent, namely, 15 mg·L^−1^ for N, 2 mg·L^−1^ for P, and 125 mg·L^−1^ for COD [22]. 

A study with two constructed wetlands using different plant species (*Vetiveria zizanioides* and *Cyperus alternifolius*) determined variations in removal of contaminants and organic matter concentrations in pig farm wastewater according to the season. The removal efficiencies were between 70% and 80% in spring, with a hydraulic retention time (HRT) of 1–2 days. The COD removal efficiency in summer reached 90%, with a COD inflow concentration of 1000–1400 mg·L^−1^. COD and BOD removal in autumn was between 50% and 60%, with HRT of 1–2 days, while in winter COD removal was 70% when the COD concentration in the influent was 1003 mg·L^−1^. There were no significant differences between the two wetlands in terms of COD and BOD removal [23]. Another research optimized constructed wetlands by using 3- and 7-day HRTs to treat wastewater from a pig farm. Seven days of HRT resulted in removal efficiencies of about 100% for total phosphorous (TP) and COD, 94.0% (NH_4_–N), and 36.6% (NO_3_) [24]. Another study set up four wetlands to assess different hydraulic nutrient loads. The system was efficient in removing organic matter, ammonia-nitrogen, nitrates, and phosphorous. However, one of the main obstacles was the low level of ammonia-nitrogen removal, especially with a high flow rate (>100 m^3^/ha/day). Nitrification was also higher in summer than in winter. Recycling the water in the process increased ammonia-nitrogen removal, but the cost of the operation also increased [25]. According to other authors [26], a subsurface constructed wetland for treating pig farm wastewater at the La Salada Renewable Resources Center in Colombia obtained removal efficiency of over 80% for organic matter and 90% for both nitrogen and phosphorous. In the case of Mexico, there have been reported removal efficiencies of 52–78% for COD, 22%, 57–79% for total nitrogen and 63% and 75% for ammonium nitrogen using subsurface flow constructed wetlands for treating pretreated swine wastewater [20]. In Mexico, the substrate material most frequently used as filter medium in constructed wetlands is the red volcanic rock, commonly named “tezontle”. The authors stated that tezontle characteristics, such as apparent porosity/void space (56%), the particle diameter; d_10_ (0.48 mm), d_60_ (1.9 mm), and average pore diameter (26.6 nm), are in the recommended range to be used as a filtration medium in constructed wetlands [27]. 

Because pig farms are continually growing in the study area, it is necessary to have low-cost ecological alternatives to treat wastewater. The objective of this research was to evaluate an in-series constructed wetland to remove organic matter and nutrients from wastewater generated by a pig farm. Our hypothesis was that the wetlands and the sedimentation tank can be connected in a series so that wastewater can flow through the three systems, which can increase the capacity of the system to remove COD, TKN, NH_3_–N, TP, and total dissolved solids (TDS) from wastewater from pig farms. The evaluation of the system seeks to provide information regarding to the system’s efficiency in removing organic matter and nutrients under the local environmental conditions.

## 2. Materials and Methods

This research was carried out on the Santa Maria pig farm, located 11 km northeast of the city of Arandas in Jalisco State, Mexico, at 20°45′35.05″ latitude north and 102°25′58.17″ longitude west, at an average altitude of 2026 a.m.s.l. (above mean sea level). The average temperature in the study area is 19 °C, with a temperature variation of 7.6 °C throughout the year. Pigs are bred for slaughtering and the farm has an inventory of 12,000 pigs with some fluctuations. The farm has an anaerobic digester to treat wastewater. The digester has a capacity of 9518 m^3^ and generates approximately 2000 m^3^ of bio-gas per day. The effluent from the anaerobic digester goes to an artificial lagoon where it is stored and used to irrigate pastures. The wastewater stored in the lagoon diluted with well water was the influent in this study.

**Figure 1:**
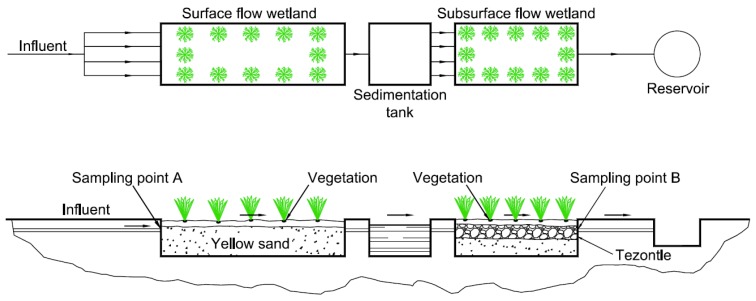
Constructed wetland in series.

The wetland in this research was a combination of systems. The first cell was a horizontal surface flow wetland (HSFW) 6 m long and 2 m wide with 5% of slope, composed of a 30 cm layer of a mixture of sand and clay (commonly called yellow sand) (the diameter ranged from 2 to 8 mm), which served as a substrate for the vegetation. The second was a sedimentation cell 2 m long and 2 m wide, with a pit to capture solids not collected in the surface wetland. The last cell was a horizontal subsurface flow wetland (HSSFW) 4 m long and 2 m wide composed of a layer of yellow sand 25 cm thick, followed by a 10 cm thick layer of red volcanic rock (tezontle). The research group did not asses the physical characteristics of the red volcanic rock. However, other authors who have used red volcanic rock from the same area reported a total porosity of 55.5% and aeration porosity of 40.7% [28]. Total porosity reported in other studies ranges from 67% to 74.7%, with aeration porosity levels of 39.2–44.4% and real density of 2.45 g·cm^−3^ [29,30]. Other studies have reported an average pore diameter of 26.24 nm and apparent porosity/void space of 56.2% [31]. After the tezontle, another layer of sand was placed as a substrate for the vegetation. In contrast to the HSFW, the water moved within the system was in direct contact with the roots of the vegetation (*Thypa* sp. and *Scirpus* sp.). The cells were made of 4 mm thick high-density geomembrane, and after the programmed retention time had passed, the water was stored in a reservoir (Figure 1). 

### Operation of the System

Since the wastewater from the anaerobic digester and lagoon had a high COD concentration, the wastewater was diluted with well water to obtain the desired COD concentrations of 400, 800, and 1200 mg·L^−1^. The wastewater from the lagoon was pumped into a 2500 L tank with a submersible pump where the dilutions were carried in order to obtain the organic load needed for each treatment and feed the wetland. Flowmeters and valves were used to control the quantity of water needed according to the desired dilution, and the pump was turned on and off by an electrical sensor. The tank was equipped with a mechanical stirrer that was activated in accordance with the on–off cycles of the pump, which in turn were regulated with a float. The water tank was placed on the edge of the lagoon, 4 m above to the wetland to ensure the water flowed by gravity from the tank to the in-series constructed wetland (ISCW). The inflow rate was controlled by a rotameter with a ball valve operating at 0.665 L·min^−1^ and a hydraulic load of 0.9576 m^3^·d^−1^ for the 5-day HRT, while the inflow rate for the 10-day HRT was 0.333 L·min^−1^ and a hydraulic load of 0.4795 m^3^·d^−1^. Equation (1) was used to calculate the inflow rate required for each HRT. The depth of the wetland was maintained at 20 cm. Once the HRT was complete, the water passed to the sedimentation cell, then to the subsurface flow wetland, and finally to the reservoir where it was stored for subsequent use.

(1) $$\tau_{n}=\frac{V}{Q}$$

where:

*V* = volume of water in the wetland (m^3^)
*Q* = volumetric flow rate (m^3^·d^−1^)
*τ_n_* = nominal retention time (days)

There was an adaptation period of approximately four months for the system before starting the experimental phase. The vegetation density was estimated at 75%. The experiments were carried out from September 2014 to November 2016, with two research series being conducted (*n* = 96). There were six runs with six duplicates for a total of 12 experimental runs, four using 400 mg·L^−1^ of COD, four with 800 mg·L^−1^ of COD, and four with 1200 mg·L^−1^ of COD.

Water samples were collected at 5-day intervals for 5-day HRT, and at 10-day intervals for 10-day HRT, following the official Mexican standards (NMX-AA-003-1980) for water sampling. The sampling points were the inlet (point A) of the surface flow wetland and the outlet of the subsurface flow wetland (point B) (Figure 1). There was a period of 10 days at the end of each experiment for the maintenance of the system, with an effort to obtain the same conditions as at the beginning of each experiment. During these 10 days, the water flow was maintained with well water. Because of the dilutions (lagoon water mixed with well water), it was necessary to monitor continuously to ensure that the COD remained at a level required for each experiment (400, 800, and 1200 mg·L^−1^), because of which the COD was used instead of BOD_5_ as a base.

The parameters measured in the field were: temperature (°C), pH, (mg·L^−1^), electrical conductivity (EC) (µS·cm^−1^), and TDS (mg·L^−1^), using the MW 801 Milwaukee sensor (Milwaukee Instruments, Melrose, MA, USA) and dissolved oxygen (DO) was determined, using a potentiometer JPB, model 607A (Milwaukee Instruments, Melrose MA, USA). The laboratory parameters were: COD (mg·L^−1^), HACH 8000 Digestion method, TKN (mg·L^−1^) HACH 10072 Persulfate digestion method, NH_3_–N (mg·L^−1^) HACH 10031 Salicylate method, and TP (mg·L^−1^) HACH 10127 Molybdanate method with acid persulfate digestion. 

Excel was used to analyze the mean, standard deviation, minimum, and maximum of the evaluated parameters. The statistical analysis was made using a 3 × 2 factorial treatment design. Factor A was the organic matter load, with three levels of COD: 400, 800, and 1200 mg·L^−1^ (Treatment 1 = 400 mg·L^−1^, Treatment 2 = 800 mg·L^−1^, and Treatment 3 = 1200 mg·L^−1^). Factor B was hydraulic retention time and included two levels: 5 and 10 days of retention. Minitab was used to perform the statistical analysis. The quantified variables were: (a) independent variables: organic matter and HRT and (b) dependent variables: removal efficiency of COD, TP, and TDS.

## 3. Results and Discussion

Table 1 shows the mean, standard deviation, minimum, and maximum of the influent, effluent, and removal efficiency for COD, TKN, NH_3_–N, TP, and TDS in the three treatments (T1 = 400, T2 = 800, and T3 = 1200 mg·L^−1^), based on 5 days (5-day HRT) and 10 days (10-day HRT) of HRT. Table 2 shows the mean, standard deviation, minimum, and maximum of the influent and effluent for the in situ parameters. Temperature, pH, DO, and EC in the three treatments (T1 = 400, T2 = 800, and T3 = 1200 mg·L^−1^) were based on 5 days (5-day HRT) and 10 days (10-day HRT) of HRT.

***Water temperature.*** The average inflow–outflow temperatures for 5-day HRT ranged from 22 to 16 °C (Table 2). Meanwhile, the average inflow–outflow temperatures for 10-day HRT ranged from 22 to 18 °C. Water inflow and outflow temperature decreased at 5-day HRT in T3, with the average outflow temperature being 16 °C. Several authors have noticed that high water temperatures decrease the capacity to maintain DO at optimal levels [32,33], because of which it is important to measure temperature in the field at the same site where oxygen is measured to facilitate analyzing the correlation between the two parameters [33]. Water temperature affects DO levels, the metabolism of organisms, and aquatic vegetation photosynthesis [34,35]. The water inflow temperatures in this research are within the ranges noticed by another research [36], which permitted photosynthesis and bacterial activity. However, the average outflow temperature was 4 °C below the inflow temperature, and it has been well documented that the optimal temperature for photosynthesis and bacterial activity is between 20 and 25 °C [37,38,39].

***pH.*** The pH levels in this study were in the alkaline range of 8.2–8.4, while the level in outflow was around one unit lower (7.2) than the inflow level, which could be attributed to vegetation and bacterial activity [40]. There was generally little variability in pH values during the wetland assessment (Table 2). Certain chemical processes only occur at a specific pH level [41]. The pH is involved directly in the NH_3_–N volatilization and orthophosphate precipitation. High pH (around 9–10) values due to algae photosynthesis may increase those mechanisms important in some nutrient removals [42,43].

***Dissolved oxygen.*** Dissolved oxygen (DO) levels (0.6–2.6 mg·L^−1^) in the wastewater were below the levels necessary to support aquatic life (Table 2). Although DO decreased in all the experiments, levels in all cases remained below what is required, so that discharge of surface water represents a risk for aquatic life. The highest DO level was 2.6 mg·L^−1^. DO is a key parameter in analyzing water pollution given that it conditions aerobic and anaerobic activity in water bodies [44,45]. Plants play an important role in wetlands treatment efficiency, specifically as the plant root systems comprise a substantial part of wetland biomass. Plants mainly influence the purification process, and this effect can be observed in the DO, pH, and redox potential (ORP) activities in the areas surrounding the roots and stems. The DO reduction from wetland influent to the effluent in the present study ranged from 1.5 to 1.8 mg·L^−1^. This result suggested some oxygen consumption by the oxidation of organic matter and nitrification. 

***Electrical conductivity*.** The decrease in electrical conductivity (EC) was greater with the 10-day HRT outflow (23%) than with that of the 5-day HRT (12%). The EC ranging from 502 to 1836 µS·cm^−1^. EC measures the concentration of salts in the water, which is a parameter that strongly affects plant growth. The first effect of high EC levels on vegetation is the inability of plants to compete for ions in water and soil (physiological drought). The higher the EC, the less water is available for plants even when the soil appears to be moist [46]. The EC levels in this study do not represent a risk for vegetation growth, which was reflected in the continual growth of vegetation in the wetland throughout the experiment (Table 2).

**Figure 2:**
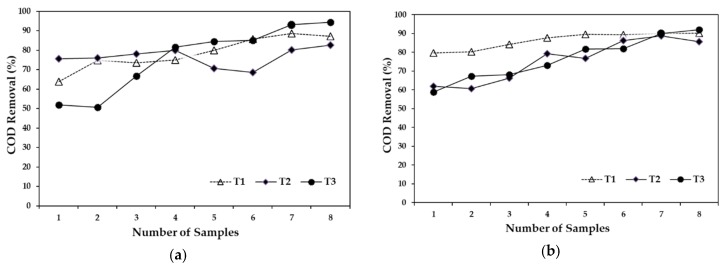
Chemical oxygen demand removal, (**a**) compares the removal efficiencies of the three treatments with a 5-day HRT and (**b**) compares the removal efficiencies of the three treatments with a 10-day HRT.

***Chemical oxygen demand.*** The 5-day HRT had the lowest removal efficiency in T2 and T3, with 76% on average. The highest removal efficiency was on T1 (79%), however, it was slightly higher than that of T2 and T3. It should be noted that the difference between the lowest and highest removal efficiency was only 3%, approximately. Figure 2a compares the removal efficiencies of the three treatments with a 5-day HRT. The removal efficiency with a 10-day HRT was 86% for T1, 76% for T2, and 77% for T3. Figure 2b shows the COD removal in the three treatments with a 10-day HRT. Removal efficiency for the three treatments presented the same tendency, with T1 increasing by approximately 10%, while T2 and T3 increased around 25%. Using an artificial wetland, other authors [47] obtained a removal efficiency of 85% of organic matter, which is similar to the removal obtained in the present study. However, the results obtained in this research are in disagreement with the results observed in others reports, where 90% of COD removal was obtained with 1–2 days of HRT during the summer season and an inflow COD concentration between 1000 and 1400 mg·L^−1^ [23]. However, results in the present study were similar to those reported by the same authors for the spring season (70–80% COD removal) and other studies in Belgium where the COD removal efficiency ranged from 64% to 75%, although the environmental conditions were different [21]. On the other hand, the results of this research are similar to those reported in Mexico using subsurface flow constructed wetlands for treating pretreated swine wastewater (COD removal efficiency of 52–78%) [20]. Several authors have stated that sedimentation played an important role in the COD removal [37,48,49]. Due to COD removal being more affected by physical forces, the effect of plant density and growth in spring followed by plant decay in fall and winter may influence the wetland performance [37].

**Figure 3:**
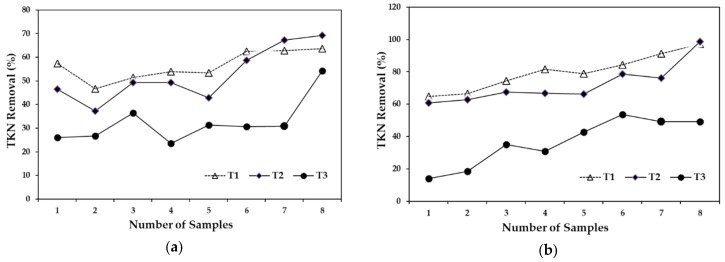
Total nitrogen removal, (**a**) compares the removal efficiencies of the three treatments with a 5-day HRT and (**b**) compares the removal efficiencies of the three treatments with a 10-day HRT.

***Total nitrogen.***Figure 3a graphically shows the TKN removal efficiency with a 5-day HRT. The removals were similar for T1 and T2 at 56% and 53%, respectively, meanwhile T3 showed removal efficiency of 32%. On the other hand, T2 and T3 had similar patterns with a 10-day HRT as with a 5-day HRT, with removal efficiency increasing by approximately 20% (Figure 3b). The removal of T1 remained stable, reaching an average of 87%, followed by T2 at 72%, and finally T3 at 37%. The efficiency in TKN removal differed with a 5- and 10-day HRT. The difference was most notable with T1, in which the 10-day HRT resulted in a 30% higher removal than that of the 5-day HRT.

The TKN removal results obtained in this research were significantly lower than the results reported by other research [13], where they achieved high removal efficiency for TKN of around 95–98%. However, in their experiments, the inflow water was from aquaculture facility effluent, and they used a subsurface and surface flow wetland combination. The results in the present study (T1 and T2 and 10-day HRT) slightly differ from the research carried out in Belgium, where they reported 83% of nitrogen removal [21]. Nevertheless, the results obtained in this study were similar to those reported in other research in Mexico, with the authors obtaining about 57–79% for TKN removal, using subsurface flow constructed wetlands for treating pretreated swine wastewater [20]. The efficiency obtained in this experiment were similar to those reported in other research in the treatment of pig farm wastewater with a surface wetland [23]. These authors used COD concentrations of between 1000 and 1400 mg·L^−1^, resulting in TKN removal efficiency of 70% in spring. The efficiency of the system increased during the summer, reaching 90%, and then decreased to 50% in autumn and winter. Notably, in the present study, only T1 with a 10-day HRT obtained a removal as high as 87%, because of which the results are below the summer efficiency levels reported by other research [23]. According to some research, nitrogen removal is more strongly influenced by season and temperature compared with removal of P because it is mainly affected by microbial activity [37]. According to this statement, the temperature could influence the results in the present study since the average air temperature in the study area was 19 °C with a temperature variation of 7.6 °C throughout the year. It has been stated that the optimal temperature for photosynthesis and bacterial activity is between 20 and 25 °C [37,38,39]. However, the temperatures in this study were within the ranges mentioned by another study, which allows for photosynthesis and bacterial activity [36]. 

**Figure 4:**
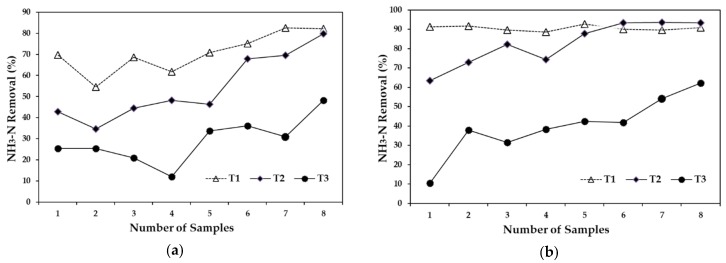
Ammonia nitrogen removal, (**a**) compares the removal efficiencies of the three treatments with a 5-day HRT and (**b**) compares the removal efficiencies of the three treatments with a 10-day HRT.

***Ammonia nitrogen.*** The NH_3_–N removal efficiency with a 5-day HRT was 71% for T1, 54% for T2, and 29% for T3. Figure 4a compares the results of the three treatments. Variability in removal was observed, with a tendency for them to increase, in particular in T2 and T3, in which removal increased about 50%. The average NH_3_–N removal with a 10-day HRT were 91% for T1, 84% for T2, and only 40% for T3. Figure 4b compares the results of the three treatments, where it can be observed that NH_3_–N removal efficiency for T1 and T2 largely remained constant, with a slight increase in the last sampling date. In contrast, T3 had the lowest removal efficiency, as well as presenting a significant increase in the removal efficiency (10%) in the last sampling. NH_3_–N removal was generally more efficient with a 10-day HRT than with a 5-day HRT. The highest NH_3_ removal efficiency of 91% was obtained in T1, which was 20% higher than what was obtained in T1 at 5-day HRT. The difference between a 5-day and 10-day HRT for T2 was 30%, while for T3 it was approximately 20%. NH_3_–N removal efficiency of 93% using a vertical wetland and an input concentration of 459 mg·L^−1^ was reported by other researchers [50]. The highest level of efficiency was achieved in T1 (90%) with an average influent inflow of 15.6 mg·L^−1^. The removal efficiency for T2 and T3 with a 10-day HRT were 84% and 40%, respectively.

Ammonia removal efficiency in the present study (T1 = 91%, T2 = 83% 10-day HRT, and 71%, 5-day HRT) differed from those in other research [51] which obtained almost 100% ammonia-N removal in a vegetated wetland system in a study using sewage in a laboratory-scale constructed wetland system. However, results of the present research (T2 = 54% with 5-day HRT and T3 = 40% with 10-day HRT) are in reasonable agreement with those of other research [52], which obtained a removal efficiency of 52%. The reduction of NH_3_–N in the present study from point A to point B of the wetland system may indicate nitrification or volatilization [43]. This condition usually occurs in aerobic environments, which is the case of surface water wetland systems. Plants prefer inorganic (nitrate and ammonium) and organic (urea and amino acids) forms of nitrogen. Nitrogen uptake may also be influenced by environmental factors such as air temperature, aeration, pH, salt concentration in the root zone, as well as the plant’s growth stage [53]. The reduction of pH and DO in the present study might be an indicator of nitrification and oxidation of organic matter. However, nitrates and nitrites were not analyzed in the present study. On the other hand, research conducted in a constructed wetland under subtropical monsoon climate conditions in Taiwan (average annual temperature of 15.5 °C) demonstrated about 56.7 kg·N·ha^−1^ and ammonia volatilization losses of about 18%. The results demonstrated that relatively shallow water and higher temperatures might strongly induce ammonia volatilization [54]. In accordance with this statement and the average air temperature (19 °C), some percentage of ammonia losses in the present study suggested some ammonia volatilization. However, in the present study, ammonia losses by volatilization were not assessed.

**Figure 5:**
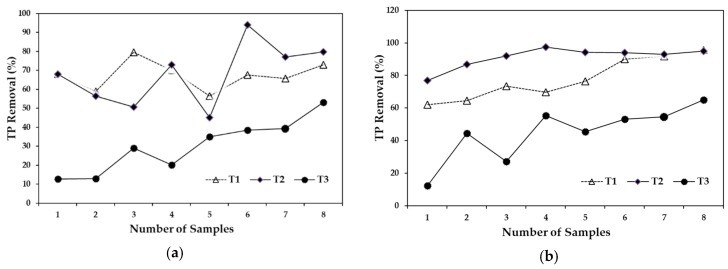
Total phosphorous removal, (**a**) compares the removal efficiencies of the three treatments with a 5-day HRT and (**b**) compares the removal efficiencies of the three treatments with a 10-day HRT.

***Total phosphorous.***Figure 5a shows the TP removal efficiency with a 5-day HRT. The efficiency for T1 and T2 were similar, at 67% and 68%, respectively. The removal for T3 was significantly lower, beginning at 50% and increasing to approximately 12%, with an average efficiency of 22%. Average TP removal efficiencies with a 10-day HRT were 88% for T1 (Figure 5b), 61% for T2, and only 45% for T3. The removal efficiencies with a 10-day HRT were higher than those obtained at a 5-day HRT, verifying that removal efficiencies are higher and more stable with longer water retention time. The efficiency of treating pig farm wastewater with an artificial wetland in Murcia, Spain reported a TP removal efficiency of 33% with four weeks of HRT. The results show that the HRT was a determining factor for the efficiency of the system [55]. The highest removal efficiency in this study was 88%, with an HRT of 10 days, which is 40% higher than what other researchers have reported [55,56]. However, it should be noted that several factors were different in the two studies, such as vegetation and retention time, as well the designs of the respective wetlands. The high removal efficiency variation between treatments in the present study made it difficult to compare the results. The results ranged from 22% at T3 with 5-day HRT to 91% in T2 with 10-day HRT. However, in the present study (T2 = 91%, 10-day HRT), the findings are in agreement with results obtained in a subsurface flow wetland, with P removal efficiency of 92% [21]. This result (T2 = 91%, 10-day HRT) is also in agreement with another study conducted in a subsurface constructed wetland for treating pig farm wastewater at the La Salada Renewable Resources Center in Colombia, with the P removal reported at about 90% [26]. It has been stated that P removal is less affected by temperature and that sedimentation and adsorption are the main processes of P removal in wetland systems [17].

**Figure 6:**
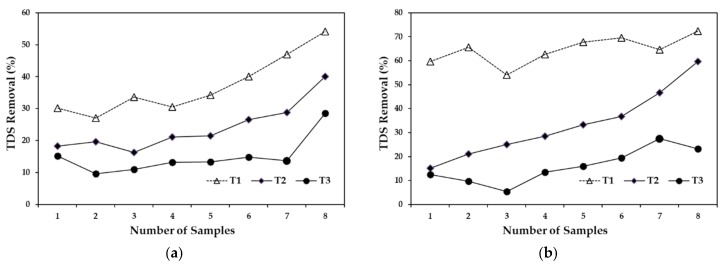
Total dissolved solids removal, (**a**) compares the removal efficiencies of the three treatments with a 5-day HRT and (**b**) compares the removal efficiencies of the three treatments with a 10-day HRT.

***Total dissolved solids.***Figure 6a shows the TDS removal efficiency at 5-day HRT. The graph shows the removal efficiency for all three treatments. It was observed that T2 and T3 tended to increase by around 20%, while there was a more notable increase of 30% in T1, resulting in an average removal of 37% for T1, 24% for T2, and only 15% for T3 with a 5-day HRT. Figure 6b also shows the removal efficiency with a 10-day HRT. The removal efficiency for T1 remained stable, with an overall average of 65%, while T2 presented the highest degree of variability, with an increase of approximately 55%. The removal for T3 was less variable but was only 16% (Figure 6b). Solids in the water basically refer to particles in suspension and dissolved particles. One of the main processes that takes place in wetlands is sedimentation, provided particles in suspension have sufficient time to settle [15]. It should be noted that another way in which wetlands reduce contamination is through pollutants adhering to particles of organic matter in suspension, so that part of the wetland efficiency is due to sedimentation rather than by absorption by vegetation [15]. The results suggest that the low TDS removal efficiency in this study was mainly due to the size of the particles in suspension, which was approximately 1.2 µm. The lowest TDS removal was 15% for a 5-day HRT, while the highest was 65% with a 10-day HRT.

It is well known that sedimentation is a physical process and is not highly affected by temperature [37]. Some studies also demonstrated that suspended solids may be captured in floating mats [48,49,57].

### The Effect of the Hydraulic Retention Time (HRT) and the Treatments of the Removal Efficiencys of COD, TP, and TDS

**Figure 7:**
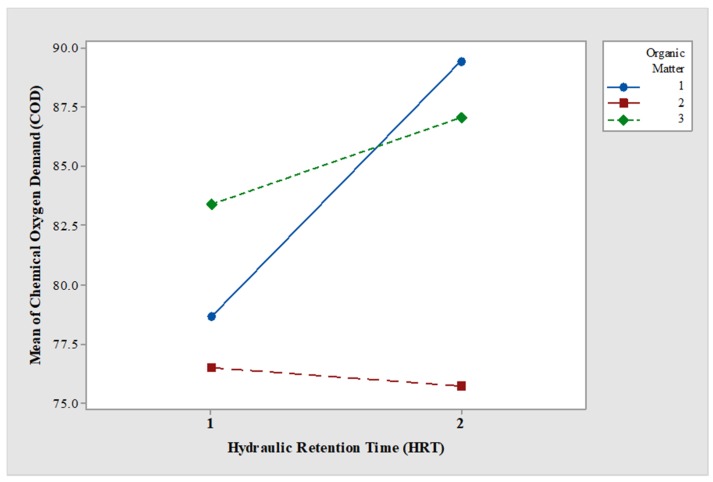
Interaction between COD concentration and HRT.

The dependent variables in the statistical analysis were the removal efficiency of COD, TP, and TDS owing to two controlled factors, namely the initial COD concentration (Factor A), with three levels (400, 800, and 1200 mg·L^−1^) and the water retention time (5 and 10 days) (Factor B). Results demonstrated significant differences in the variable COD due to factor A (*p* < 0.05) and the A–B interaction (*p* < 0.05), but not due to factor B (*p* > 0.05). The mean values of COD removal were 76% and 83% for 5-day HRT and 76–87% for 10-day HRT (Figure 7).

**Figure 8:**
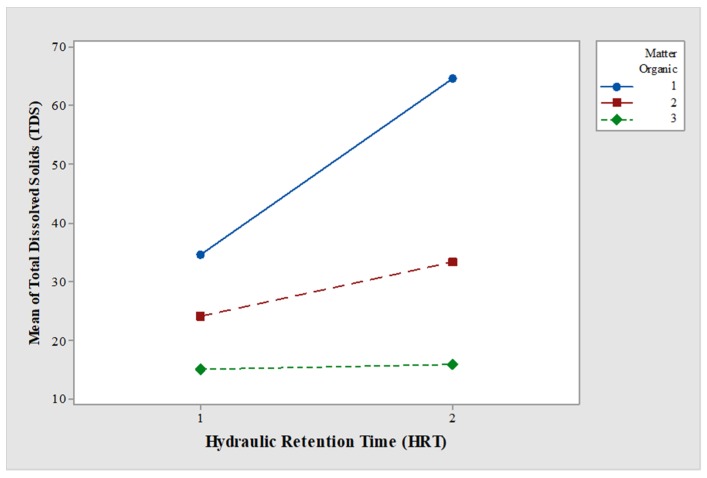
Interaction between TDS and HRT.

There were differences in the variable TP owing to factors A (*p* < 0.05) and B (*p* < 0.05), but not due to A–B interaction (*p* > 0.05). The mean values were from 32% to 68% for 5-day HRT and 10-day HRT. There were significant differences in the variable TDS owing to factors A (*p* < 0.05), B (*p* < 0.05), and A–B interaction (*p* < 0.05). The mean values were from 45% to 88% for 5-day HRT and 10-day HRT (Figure 8). Results obtained in this research demonstrated the interaction between the treatment efficiency and the retention time, and these results are in agreement with other studies [12,58] where strong dependence on retention time and wetland efficiency was reported.

According to several reports [53,54], the removal of nitrogen is more affected by temperature, therefore, the effect of HRT in the removal efficiency of TKN and NH_3_–N was not evaluated in the present study. 

The concentrations in outflow in the context of the different parameters were compared to the maximum permitted limits under Mexican legislation (NOM-001-SEMARNAT-1996, Mexico City, Mexico). The levels of concentrations comply with NOM-001 for using water for irrigation, but it is desirable that the initial level of COD is not greater than 200 mg·L^−1^ and that the treatment of pig farm outflow involves a 10-day HRT to ensure efficient removal of contaminants. The NOM-001 required 40 mg·L^−1^ of TKN, 20 mg·L^−1^ of TP, 150 mg·L^−1^ of TSS, and 150 mg·L^−1^ of BOD_5_. 

## 4. Conclusions

Given the low cost of installing and maintaining artificial wetlands in series, they represent a viable option for treating pig farm wastewater and removing COD, TKN, NH_3_–N, TP, and TDS, with an average removal efficiency of higher than 70%. Results demonstrated that *Thypa* sp. and *Scirpus* sp. are a suitable macrophyte species to be used for the pig farm wastewater treatment under local environmental conditions. It is highly recommended to continue sampling as the in-series constructed wetland continues to mature in order to determine the nutrient storage soil capacity and the nutrient dynamics under long-term treatment and different hydraulic retention time. It would be also important to determine the rate of denitrification, nitrification, and ammonia volatilization under different weather conditions. It is suggested to perform the material balance in future work and determine the main pathways for the removal of pollutants.

One of the main challenges that was detected is the removal of TDS. Since the particle size is very small, it is difficult to settle in the wetland. It is necessary to make adjustments in the system to increase the removal of pollutants and comply with the official regulations necessary to use the effluent water as irrigation water. In this context, the Mexican legal standard indicates that the organic matter content (BOD_5_) should not exceed the maximum allowed limit of 150 mg·L^−1^ applicable for agricultural irrigation. There is no maximum permissible limit for COD in Mexico. However, given that BOD_5_ is equivalent to 1.6 times the organic matter content expressed by COD [59], the organic matter concentration of the effluent complied with the Mexican standards.

Even though the in-series wetland has great potential for pig farm wastewater treatment, it is also important to mention that a family farm in the region generates around 6000 L·d^−1^ of wastewater, therefore a wetland with an area of 160 m^2^ with a HRT of 5 days to obtain acceptable removal efficiency is required. Therefore, future studies should explore alternatives to improve the wetland performance.

## Figures and Tables

**Table 1:** Mean, standard deviation, minimum, and maximum of influent and effluent concentration and removal efficiency based on the evaluated parameters.

**400 mg·L^−1^**															
	**COD**			**TKN**			**NH_3_–N**			**TP**			**TDS**		
**5-day HTR**	**Ci**	**Co**	**RE (%)**	**Ci**	**Co**	**RE (%)**	**Ci**	**Co**	**RE (%)**	**Ci**	**Co**	**RE (%)**	**Ci**	**Co**	**RE (%)**
mean	454	97.2	78.6	44.3	19.1	56.5	32.2	9.40	70.7	12.7	4.10	67.4	452	288	37.1
sdv	15.0	38.0	8.30	5.00	2.50	6.10	2.20	2.90	9.60	1.50	0.80	7.04	100	94.3	9.31
min	430	53.0	64.0	35.0	16.0	46.7	29.5	5.50	54.5	11.7	3.30	56.6	315	195	27.1
max	480	165	88.5	51.0	24.0	63.6	35.0	14.2	82.5	16.4	5.30	79.6	610	426	54.1
**10-day HRT**	**Ci**	**Co**	**RE (%)**	**Ci**	**Co**	**RE (%)**	**Ci**	**Co**	**RE (%)**	**Ci**	**Co**	**RE (%)**	**Ci**	**Co**	**RE (%)**
mean	413	56.2	86.3	30.6	6.3	79.8	15.5	1.50	90.5	10.2	2.20	77.9	357	126	64.5
sdv	9.90	17.6	4.30	5.70	4.20	11.2	1.20	0.20	1.40	0.70	1.30	12.9	23.5	25.0	5.81
min	394	40.2	79.8	22.8	1.00	64.8	13.1	1.10	88.5	9.60	0.50	62.2	320	98.0	54.1
max	425	84.0	90.1	39.2	12.8	97.1	17.1	1.70	92.8	11.5	4.21	95.7	401	170	72.4
**800 mg·L^−1^**															
	**COD**			**TKN**			**NH_3_–N**			**TP**			**TDS**		
**5-day HRT**	**Ci**	**Co**	**RE (%)**	**Ci**	**Co**	**RE (%)**	**Ci**	**Co**	**RE (%)**	**Ci**	**Co**	**RE (%)**	**Ci**	**Co**	**RE (%)**
mean	810	185	76.5	94.4	45.0	52.6	66.3	30.4	54.2	20.5	6.4	68.0	591	447	24.0
sdv	117	12.6	4.80	15.3	14.7	11.5	10.6	12.5	15.9	4.60	3.20	16.4	76.2	61.9	7.71
min	69.0	28.0	37.3	69.0	28.0	37.3	48.3	13.5	34.6	13.6	1.20	45.1	490	390	16.3
max	114	69.0	69.2	114	69.0	69.2	77.4	48.6	79.7	26.0	10.6	94.0	710	580	40.0
**10-day HRT**	**Ci**	**Co**	**RE (%)**	**Ci**	**Co**	**RE (%)**	**Ci**	**Co**	**RE (%)**	**Ci**	**Co**	**RE (%)**	**Ci**	**Co**	**RE (%)**
mean	740	177	75.7	71.6	19.7	72.2	35.9	6.51	82.6	17.2	1.51	91.1	49.0	329	33.3
sdv	89.4	79.0	11.3	10.0	9.20	12.4	5.50	4.41	11.4	2.10	1.00	6.60	34.6	66.7	14.4
min	607	87.0	60.6	56.0	1.00	60.7	26.0	1.70	63.5	14.5	0.40	76.8	450	226	15.2
max	869	299	88.7	89.0	29.0	98.7	42.7	13.4	93.5	19.9	3.52	97.5	560	410	59.6
**1200 mg·L^−1^**															
	**COD**			**TKN**			**NH_3_–N**			**TP**			**TDS**		
**5-day HRT**	**Ci**	**Co**	**RE (%)**	**Ci**	**Co**	**RE (%)**	**Ci**	**Co**	**RE (%)**	**Ci**	**Co**	**RE (%)**	**Ci**	**Co**	**RE (%)**
mean	1247	296	76.0	131	88.0	32.5	97.0	67.9	29.1	32.6	22.8	30.1	918	781	14.9
sdv	57.3	205	17.4	13.5	15.5	9.60	11.2	7.70	10.9	2.10	4.60	14.2	50.0	62.0	5.81
min	1153	68.0	50.7	116	54.0	23.6	82.3	52.6	12.0	30.0	14.3	12.7	830	650	9.60
max	1339	578	94.5	154	102	54.2	111	78.0	48.3	35.8	28.0	53.1	1010	860	28.6
**10-day HRT**	**Ci**	**Co**	**RE (%)**	**Ci**	**Co**	**RE (%)**	**Ci**	**Co**	**RE (%)**	**Ci**	**Co**	**RE (%)**	**Ci**	**Co**	**RE (%)**
mean	1089	537	76.6	112	69.3	36.7	84.4	49.9	39.8	27.7	14.7	44.7	786	657	15.9
sdv	147	154	11.7	18.2	9.50	14.7	9.70	9.50	15.4	4.40	2.30	17.1	81.4	48.3	7.21
min	814	65.0	58.9	78.0	52.0	14.1	67.2	33.2	10.4	19.4	10.8	12.4	640	560	5.31
max	1232	500	92.0	130	83.0	53.6	101	60.2	62.2	31.9	17.9	65.0	910	710	27.5

Ci = influent concentration, Co = effluent concentration, RE (%) = removal efficiency.

**Table 2:** Mean, standard deviation, minimum, and maximum of influent and effluent based on the evaluated parameters in situ.

**400 mg·L^−1^**								
	**Temperature (°C)**		**pH**		**DO mg·L^−1^**		**CE µS·cm^−1^**	
**5-day HTR**	**in**	**out**	**in**	**out**	**in**	**out**	**in**	**out**
mean	22.0	19.9	8.2	7.20	0.68	2.14	629	502
sdv	0.60	0.60	0.10	0.10	0.12	0.31	72.3	50.0
min	21.1	18.9	8.10	7.10	0.50	1.80	540	430
max	22.8	21.0	8.3	7.30	0.80	2.60	712	600
**10-day HRT**	**in**	**out**	**in**	**out**	**in**	**out**	**in**	**out**
mean	22.1	21.4	8.14	7.16	0.94	2.51	637	503
sdv	1.90	2.00	0.09	0.07	0.28	0.98	78.0	50.0
min	20.0	19.4	8.00	7.10	0.40	2.00	540	430
max	25.9	26.0	8.30	7.30	1.40	3.10	720	600
**800 mg·L^−1^**								
	**Temperature (°C)**		**pH**		**DO mg·L^−1^**		**CE µS·cm^−1^**	
**5-day HTR**	**in**	**out**	**in**	**out**	**in**	**out**	**in**	**out**
mean	22.0	19.9	8.20	7.30	0.71	1.20	1170	937
sdv	0.60	0.60	0.10	0.10	0.10	0.50	155	128
min	21.1	18.9	8.10	7.10	0.50	0.60	970	780
max	22.8	21.0	8.30	7.50	0.80	2.00	1410	1170
**10-day HRT**	**in**	**out**	**in**	**out**	**in**	**out**	**in**	**out**
mean	22.1	21.4	8.14	7.21	0.90	2.10	1008	655
sdv	1.90	2.00	0.07	0.10	0.25	0.72	89.0	160
min	20.0	19.4	8.00	7.10	0.70	1.40	900	350
max	25.9	26.0	8.20	7.40	1.40	3.50	1140	830
**1200 mg·L^−1^**								
	**Temperature (°C)**		**pH**		**DO mg·L^−1^**		**CE µS·cm^−1^**	
**5-day HTR**	**in**	**out**	**in**	**out**	**in**	**out**	**in**	**out**
mean	19.4	16.0	8.50	7.70	0.58	0.44	1836	1718
sdv	1.91	1.92	0.11	0.20	0.20	0.32	92.1	185
min	16.7	13.8	8.40	7.50	0.30	0.10	1660	1300
max	21.7	18.5	8.60	8.00	0.90	0.90	1990	1890
**10-day HRT**	**in**	**out**	**in**	**out**	**in**	**out**	**in**	**out**
mean	20.7	17.7	8.25	7.28	0.89	0.98	1573	1395
sdv	1.31	1.80	0.05	0.13	0.28	0.24	181	90.1
min	19.1	15.4	8.20	7.10	0.50	0.60	1290	1270
max	22.8	20.4	8.30	7.50	1.40	1.40	1910	1520

in = influent values, out = effluent values.
